# Frequency-comb-referenced multiwavelength interferometry for high-precision and high-speed 3D measurement in heterogeneous semiconductor packaging

**DOI:** 10.1515/nanoph-2024-0578

**Published:** 2025-02-07

**Authors:** Jun Hyung Park, Dae Hee Kim, Huy Hoang Chu, Ji Won Hahm, Guseon Kang, Dongil Lee, Seyong Song, Mingu Kang, Seung-Woo Kim, Young-Jin Kim

**Affiliations:** Department of Mechanical Engineering, Korea Advanced Institute of Science and Technology, 291 Daehak-ro, Daejeon, Republic of Korea; IntekPlus Co., 263, Techno 2-ro, Yuseong-gu, Daejeon, Republic of Korea

**Keywords:** frequency comb, heterogeneous integration, microbump, 3-D profile measurement, multiwavelength interferometry

## Abstract

As Moore’s law approaches its physical limits, the semiconductor industry has begun to focus on improving I/O density and power efficiency through 2.5D/3D packaging. Heterogeneous integration, which combines integrated circuit blocks from different linewidth processes into a single package, is central to these developments. To ensure stable connections with high yield in the back-end processes, high precision and high speed 3D surface measurement is the prerequisite. Existing methods such as white-light interferometry and confocal microscopy face challenges in balancing resolution, speed, and accuracy in 3D measurements. Here, we report a frequency-comb-referenced multiwavelength interferometry for the measurement of 3D sample profiles without 2π phase ambiguity for advanced packaging. Using four frequency-comb-referenced wavelengths with a fractional stability of 4.77 × 10^−12^, the measurement range was extended from ∼400 nm (*λ*/2) to 1 mm, with the measurement repeatability of 0.258 nm for 32 measurements. The standard step-height samples with 500-µm and 4.5-µm steps, as well as real industrial microbumps in heterogeneous integration packaging, were all successfully measured. Therein, we devised a sequential phase detection method, which enables 5,000 times faster solution determination than the traditional recursive excess fraction method, while maintaining its reliability under noisy conditions. As 2.5D/3D packaging architectures become increasingly complex, our approach will readily meet the critical industrial demands for high-precision and high-speed measurement of multiscale features in advanced semiconductor packaging.

## 1 Introduction

**Figure 1:**
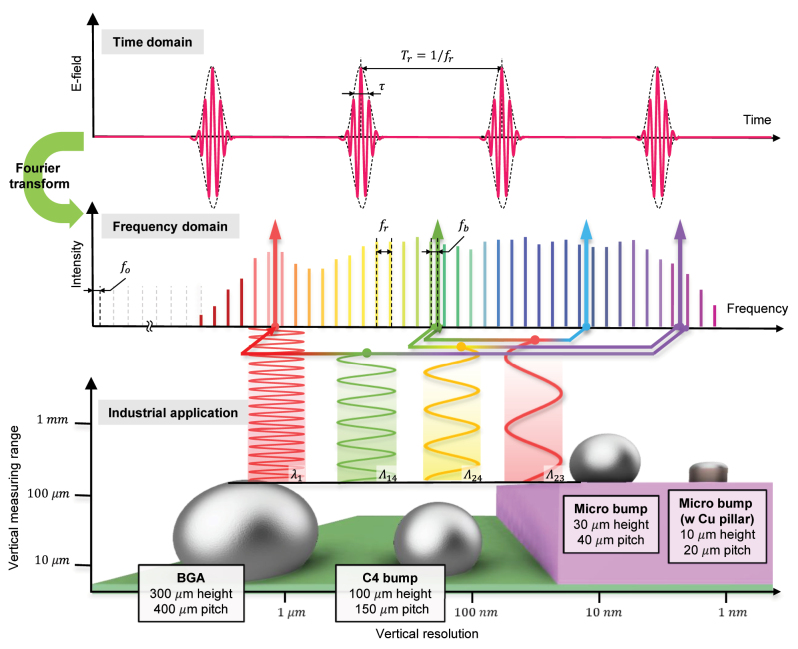
A conceptual figure illustrating the Fourier relation of a femtosecond laser-based frequency comb. In the time domain, the femtosecond laser outputs a periodic train of pulses, each with a fs-pulse duration (*τ*) and a specific pulse-to-pulse time spacing (*T*_*r*_). In the frequency domain, the modes of optical frequency comb are defined by the offset frequency (*f*_*o*_) and integer multiples of the repetition rate (*f*_*r*_), expressed as *v* = *N* × *f*_*r*_ + *f*_*o*_. Four wavelengths, phase-locked to an optical frequency comb via beat frequency (*f*_*b*_), are used to measure various interconnects in the semiconductor packaging, ensuring the vertical range and resolution requirements are met for 3D measurements.

During the era of Moore’s law, the semiconductor industry achieved device miniaturization and advancements primarily by reducing the circuit linewidths on the wafers. However, as the industry faces physical limitations in the linewidth reduction below a few nanometers, it has become evident that vertical integration through advanced packaging technologies offers a more cost-effective and competitive approach. Among packaging technologies, heterogeneous integration has emerged as a prominent solution, enabling the integration of dies fabricated at different nodes onto a single substrate. This enables the integration of diverse functions like processors, memory, sensors, and RF components, leading to improved system performance and functionality. This integration creates a semiconductor platform with a discontinuous step structure and high bump density. The microbumps, which are small-scale solder balls or metal bumps, serve as interconnections between different chips or components within the package. These bumps act as contact points for the electrical connections, allowing signals and power to pass between the chips. The size of bumps is typically in the range of a few tens to a few hundred micrometers in height, depending on the specific application and packaging technology. To achieve wide bandwidth and high power efficiency, the pitch and height of interconnects are decreasing, requiring measurement systems with higher resolution and precision over a large vertical measurement range. The bottom part of Figure 1 illustrates various types of bumps used in semiconductor packaging, which require measurement system with high vertical resolution and a wide vertical range as feature sizes decrease.

Metallic bumps are bonded to each other through a reflow process after establishing mechanical contact. Nondestructive inspection methods, such as ultrasound, X-ray, and terahertz waves, offer low resolution, making it challenging to detect fine defects accurately. Therefore, the most effective approach at present is to measure the 3D external bump shapes with high precision. To meet these key measurement requirements in heterogeneous semiconductor packaging, various measurement methods have been introduced. Due to its nanometer resolution, optical interferometry has long been used for high-precision measurement applications, such as optical testing, astrophysics, biomedical imaging, and semiconductor industries. However, in the cases where the sample height – optical path difference (OPD) between the target surface and the reference mirror – exceeds half the wavelength of the light, a single wavelength interferometer faces the critical phase ambiguity problem due to the periodic nature of light. This issue prevents a precise reconstruction of the discontinuous surface structures like the semiconductor microbumps. To overcome this limitation during the measurement of discontinuous step heights, white-light scanning interferometry (WSI) [1], [2], spectrally resolved interferometers [3], [4], and multiwavelength interferometers have been utilized [5]. In WSI, a large number of optical interferograms should be acquired over a long OPD scanning for large step-height measurement, which restricts the improvement of the measurement speed. WSI is limited in covering a large field of view due to the low spatial coherence of white light sources. Meanwhile, the spectrally resolved interferometry offers a moderate measurement resolution but requires slow lateral scanning, and its measurement range is restricted by the spectral bandwidth of the light source and the spectral resolution of the spectrometer in use. Multiwavelength interferometry (MWI) enables coverage of large lateral areas and depth ranges without mechanical scanning but requires highly stable light sources and time-consuming computations for 3D profile reconstruction [6].

A frequency comb of a femtosecond pulse laser has gained higher attention as a light source in various metrology fields due to its key advantages in high frequency/wavelength stability, and high spatio-temporal coherence over a wide spectral bandwidth [7], [8], [9], [10], [11]. Numerous measurement techniques have been developed utilizing the characteristics of femtosecond lasers, including synthetic wavelength interferometry [12], [13], unequal path interferometry [14], [15], [16], dispersive interferometry [17], [18], dual comb interferometry [19], [20], [21], and time-of-flight method [22], [23], [24], [25]. In particular, high frequency/wavelength stability is the key to enabling the step-height measurement with the best precision in step-height measuring MWI [26], [27], [28]. Recently, multi-cascade linked synthetic wavelength digital holography using optical combs has been developed [29], [30]. However, due to the use of an InGaAs CCD and optical components operating in the 1,550 nm band, limitations in lateral resolution and field of view (FOV) persist. Additionally, using spatial carrier-based phase extraction degrades the precision.

In MWI profilometry, the high phase noise caused by unstable optical wavelength and disturbances directly leads to height measurement errors. These errors result in the failure of height reconstruction due to multiple solution problems, often caused by local minima. To cope with this problem, there was a pioneering research work by the authors on MWI by introducing a frequency comb as the calibration light source [31]. Although the technological potential was demonstrated, the industrial demands on high precision and high speed in the measurement could not be attained; the low optical power, higher spatial modes, and slow data processing were the key issues. The optical power at the interferometry was only a few mW due to the power conversion efficiency of 1.25 % because the previous work used a series of near-infrared continuous-wave lasers at ∼1,560 nm and free-space second harmonic generation (SHG) unit for the wavelength conversion to the visible wavelengths. SHG system was also alignment-sensitive and could not guarantee the perfect TEM_00_ spatial mode. Therefore, there have been strong demands on higher optical power at multiple wavelengths with high wavelength stability together with the high-level alignment stability. The MWI algorithm should be also improved by multiple orders of magnitudes for real-time semiconductor inspection applications.

In this study, we report a frequency-comb-referenced MWI for realizing high-precision and high-speed surface measurement for heterogeneous semiconductor inspection. By stabilizing continuous-wave tunable lasers to a frequency comb, as illustrated in the upper part of Figure 1, we achieved four highly stabilized light sources with fractional stability of ∼10^−12^. Optimally selected combination of the wavelengths reduced the possibility of local solutions during the height determination stage, which resulted in the further extension of the measurement range. Moreover, we devised a noniterative fringe order determination method for MWI. This proposed method, a hybrid of the excess fraction method and the beat wavelength method, offers a high reliability comparable with the excess fraction method under phase noise and is fast enough for real-time acquisition and in-line processing. To evaluate our system, a 500 µm gauge block, a 4.5 µm standard height sample, and real industrial microbumps with 30 µm and 10 µm bump heights were measured. The final precision of the system was evaluated to 1.35 nm in standard deviation and 0.258 nm in Allan deviation with 32 measurement averaging.

## 2 Frequency-comb-referenced multiwavelength interferometry

**Figure 2:**
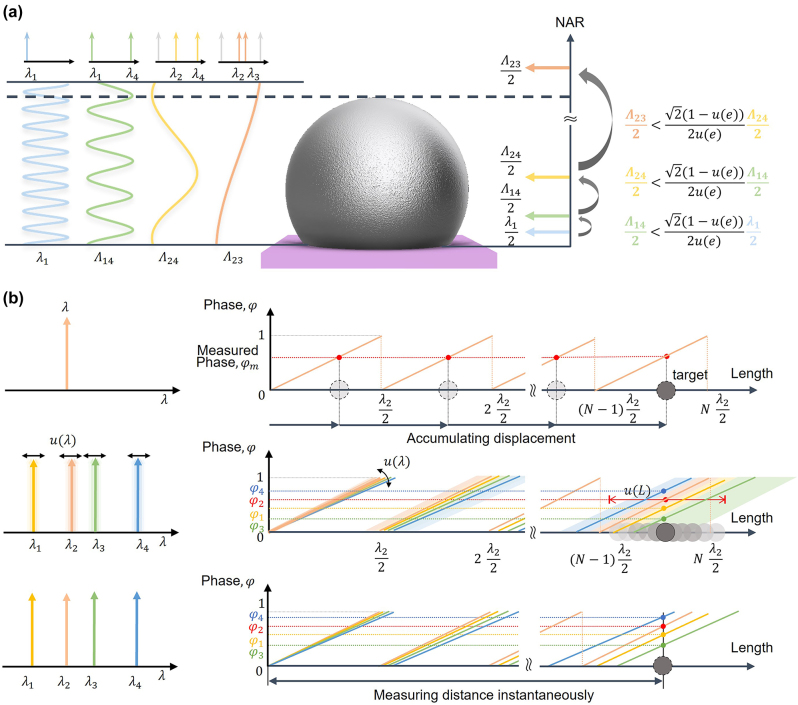
Conceptual diagram illustrating the use of multiple wavelengths for microbump measurement. (a) Magnification of the NAR by the chain rule of the synthetic wavelengths, considering phase uncertainty. (b) Length uncertainty caused by the instability of multiple wavelengths.

To measure discontinuous surfaces by MWI, an optimal set of wavelengths should be selected to ensure that the nonambiguity range (NAR) is longer than the measurand. In this study, we performed a sequential extension of the NAR, with the following wavelength selection criteria: To stably extend the NAR under noise condition, the beat wavelength generated by two wavelengths should be smaller than the phase uncertainty of the previous NAR. The NAR can be extended from half of the single wavelength to half of the largest beat wavelength as depicted in Figure 2a. Additionally, Figure 2b illustrates that phase and wavelength uncertainties can lead to multiple solutions, where local minima are incorrectly selected as the solution. This issue can be addressed by using highly stabilized light sources.

### 2.1 Optimal selection of four wavelengths for multiwavelength interferometry

An optical interferometer produces interference fringes due to the difference in optical path length between a reference plane and a measurement plane. The interference fringe, *I*, can be expressed as follows:

(1) $$\begin{array}{ll} I & =I_{r}+I_{m}+2\sqrt{I_{r}I_{m}}\cdot \cos\left\{2k\cdot L-\Delta \phi \left(k\right)\right\}\\ & =I_{o}\left[1+\gamma \cdot \cos\left\{\phi \left(x,y\right)\right\}\right] \end{array}$$

where *I*_*r*_, *I*_*m*_, and *I*_*o*_ denote the intensity reflected by the reference plane, the measurement plane, and the background intensity, respectively. *k* = 2*π*/*λ* is the propagation constant of the monochromatic light source, *L* is the length of the measurand, *γ* represents the visibility of the interference pattern, Δ*ϕ* denotes the reflection phase difference between the reference and measurement surface, and $\phi \left(x,y\right)$ represents the extracted phase. To extract the phase from the intensity formula with three unknowns – *I*_*o*_, *γ*, and $\phi \left(x,y\right)$ – at least three different equations are needed to solve the inverse problem. A phase-shifting interferometer introduces specific phase shifts, and a phase extraction algorithm resolves the arc-tangent function using the orthogonality between these phase shifts [32]. In this study, self-calibrating phase-shifting algorithms were employed to compensate for errors caused by the imperfect phase shifting of the PZT or vibrations [33], [34].

The extracted phase is nonunique due to the periodicity of phase (2π-ambiguity). In the measurement of a continuous surface, the phase can be unwrapped based on the continuity of the surface. However, for the measurement of discontinuous surfaces such as semiconductors, the measurement range is limited by the NAR. The NAR corresponds to half the wavelength of the light source for monochromatic interferometers. Multiwavelength interferometers utilize two or more wavelengths as light sources. Using multiple wavelengths allows for the extension of the NAR up to half of the largest synthetic wavelength due to different periodicities between the wavelengths [35]. In the phase extraction process, the phase for each wavelength is extracted to satisfy the following condition:

(2) $$L=\frac{\lambda_{1}}{2}\left(m_{1}+e_{1}\right)=\frac{\lambda_{2}}{2}\left(m_{2}+e_{2}\right)=\ldots =\frac{\lambda_{n}}{2}\left(m_{n}+e_{n}\right)$$

when the distance (*L*) is measured using the i-th wavelength (*i* = 1, 2, …, *n*), it is divided into the integer part (*m*_*i*_) of the phase and the fraction part (0 ≤ *e* < 1). In the case of the fraction part, *e*_*i*_, it can be obtained from solving the inverse problem of interference fringe, but the integer part m_i_ cannot be specified. The phase unwrapping algorithm of multiwavelength interferometers reconstructs the shape information (integer part) from the phase information (fractional part) of multiple wavelengths. The phase unwrapping algorithm is discussed in detail in Section 3.

Assuming a multiwavelength interferometer using two wavelengths, the measurement using wavelengths *λ*_1_ and *λ*_2_ is equivalent to that measured by the synthetic wavelength Λ_12_, and the distance *L* can be represented as follows:

(3) $$\begin{array}{ll} & L=\frac{\Lambda_{12}}{2}\left(M_{12}+E_{12}\right),\\ & \left(M_{12}=m_{1}-m_{2},E_{12}=e_{1}-e_{2},\Lambda_{12}=\frac{\lambda_{1}\lambda_{2}}{\lambda_{1}-\lambda_{2}}\right) \end{array}$$

where *M*_12_ and *E*_12_ represent the integer and fractional part of the synthetic wavelength Λ_12_. Therefore, the NAR, which can measure the step difference regardless of the integer part *M*_12_, has been extended to half of the synthetic wavelength. Here, the synthetic wavelength, Λ_12_, is inversely proportional to the difference between the two wavelengths *λ*_1_ and *λ*_2_. If two wavelengths are close enough, the NAR becomes significantly larger. However, as the synthetic wavelength increases, the phase uncertainty also increases, which can lead to local solutions for the fringe order [6], [36], [37]. Therefore, for improving measurement reliability in sub-mm step heights using near-infrared light sources, selecting more than three wavelengths while considering phase uncertainty can be beneficial [38]. Distance uncertainty *u*(*L*) caused by phase uncertainty due to wavelength instability and environmental changes should be considered to ensure that only a single integer part can become the solution. Ensuring that Equations (4)–(6) are satisfied.

(4) $$2u\left(L_{12}\right)<\lambda_{1}-2u\left(L_{1}\right)$$

(5) $$u\left(L_{1}\right)=\frac{\lambda_{1}}{2}u\left(e_{1}\right)\approx \frac{\lambda_{1}}{2}u\left(e\right)$$

(6) $$\begin{array}{ll} u\left(L_{12}\right) & =\frac{\Lambda_{12}}{2}u\left(e_{12}\right)=\frac{\Lambda_{12}}{2}u\left(e_{1}-e_{2}\right)\\ & =\frac{\Lambda_{12}}{2}\sqrt{u{\left(e_{1}\right)}^{2}+u{\left(e_{2}\right)}^{2}}\approx \frac{\sqrt{2}}{2}u\left(e\right)\Lambda_{12} \end{array}$$

Equations (4)–(6) define the conditions for using two wavelengths, ensuring no multiple solutions occur. These conditions can be expressed as:

(7) $$\frac{\Lambda_{12}}{2}<\frac{\sqrt{2}\left(1-u\left(e\right)\right)}{2u\left(e\right)}\frac{\lambda_{1}}{2}$$

To extend the NAR using four wavelengths, the extension conditions applied for two wavelengths are equally applied to the synthetic wavelengths, as described in Eq. (8).

(8) $$\frac{\Lambda_{14}}{\lambda_{1}}=\frac{\Lambda_{24}}{\Lambda_{14}}=\frac{\Lambda_{23}}{\Lambda_{24}}<\frac{1-u\left(e\right)}{\sqrt{2}u\left(e\right)}\approx \frac{1}{\sqrt{2}u\left(e\right)}$$

As described in Eq. (9), the expanded uncertainty $U\left(e\right)$ is expressed as a multiple of the coverage factor *k* and the standard uncertainty $u\left(e\right)$ . The phase uncertainty criteria between the *i*-th synthetic wavelength Λ_*i*_ and the subsequent (*i* + 1)-th synthetic wavelength Λ_*i*+1_ can be rewritten as shown in Eq. (10).

(9) $$U\left(e\right)=k\cdot u\left(e\right)$$

(10) $$\frac{\Lambda_{i+1}}{\Lambda_{i}}<\frac{1}{\sqrt{2}U\left(e\right)}=\frac{1}{k\cdot \sqrt{2}u\left(e\right)}=\frac{2\pi }{k\cdot \sqrt{2}u\left(\phi \right)}$$

Finally, with *k* = 6, corresponding to a confidence level of 99.999 999 8 %, and under the condition $u\left(e\right)≅0.004$ , the NAR was stably extended to 1 mm by selecting only four wavelengths: 777.4289 nm, 781.0084 nm, 781.3046 nm, and 786.3123 nm. However, simply using more wavelengths does not necessarily guarantee high reliability in phase unwrapping. While additional wavelengths may help determine the integer part of the phase, nm-level precision ultimately depends on the phase extraction process. Excessive use of wavelengths can be inefficient and may also increase time dependencies and environmental sensitivities, such as vibrations and drifts, which can negatively affect precision.

### 2.2 Generation of frequency-comb-referenced multiple wavelengths

Distributed Bragg reflector (DBR) type laser diode with a 3 MHz (0.006 pm @785 nm) linewidth was used, and the current of the laser diode was controlled by a feedback signal to stabilize it with the frequency comb. A femtosecond laser (Menlo systems, C-fiber) with 30 nm bandwidth at a 1,550 nm center wavelength, 100 MHz repetition rate, and 30 mW output power was used. It achieved a stability of 10^−12^ levels by locking the repetition rate, *f*_*r*_ to the Rb clock and the offset frequency, *f*_*O*_ to the optical clock. We employed SHG in order to use a Si-CMOS camera as the imaging sensor. Compared to an InGaAs-CCD designed for the 1,550 nm range (fundamental light), the Si-CMOS offers higher frame rate for fast image acquisition, smaller pixel size for higher lateral resolution, lower noise, lower cost, and greater sensor stability. To stabilize the DBR laser to the second harmonic (SH) of frequency comb, the fiber-based femtosecond laser was amplified using an Er-doped fiber amplifier (EDFA). The SH of optical frequency comb with a 785 nm center wavelength was then generated with a periodically poled lithium niobate (PPLN) crystal. The generated SH frequency comb and laser diodes were combined using a polarization beam splitter and diffracted by a blazed grating. The beating signal between each laser diode wavelength and adjacent frequency comb mode became a feedback signal through a phase-locked loop to control the current of laser diodes.

**Figure 3:**
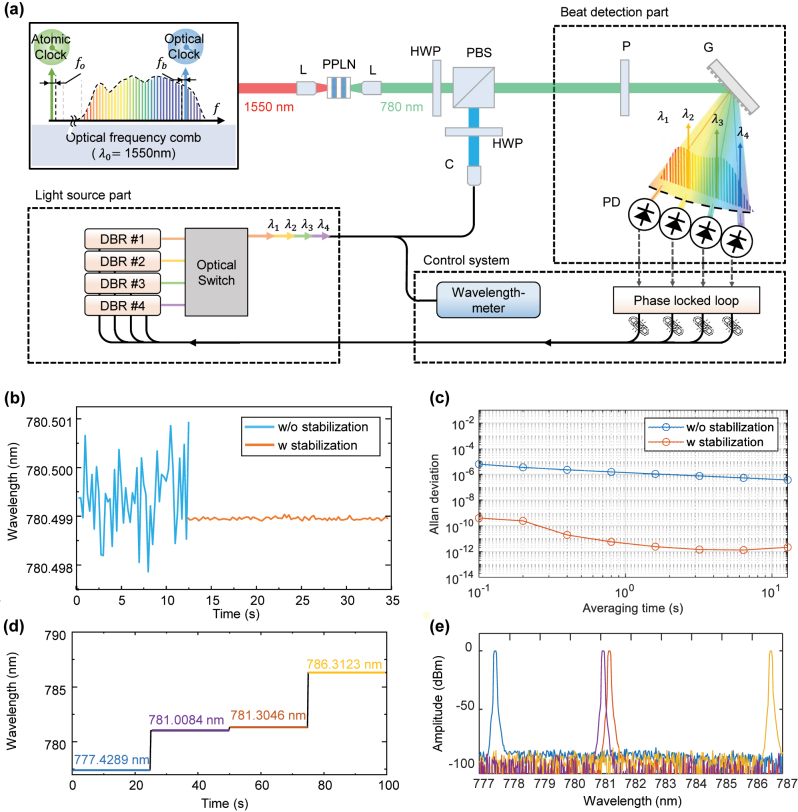
Multiwavelength interferometer based on frequency-comb-referenced laser diode. (a) Experimental set-up for stabilization of laser diode to frequency-comb. (b) Wavelength tracking w/and w/o laser diode stabilization and (c) Allan deviation. (d) Optical switching diagram and (e) spectrum of selected laser diodes. L: lens, PPLN: periodically poled lithium niobate, HWP: half-wave plate, PBS: polarization beam splitter, P: polarizer, G: grating, PD: photodetector, DBR: distributed Bragg reflector, C: collimator.

Figure 3a illustrates the configuration of the system used for phase-locking laser diodes to frequency modes of a comb. Figure 3b shows the wavelength variation before and after the stabilization of the laser diode. It was confirmed that the wavelength, which showed a 3 pm deviation before stabilization, exhibited only a 0.03 pm deviation after stabilization. Because the wavelength-meter (Bristol, CD 671) used in this study had a repeatability of 0.03 pm, measurements were limited. To accurately evaluate wavelength stability, Allan deviation is measured through a self-heterodyne interferometer. As shown in Figure 3c, the wavelength uncertainty improved by a factor of about 10^6^ times. It achieved high stability, traceable to the optical frequency comb, with an Allan deviation of 4.77 × 10^−12^ at an average acquisition time of 1 s. Figure 3d and e presents the results of optical switching over time and the spectrum of selected four wavelengths, respectively. It was generated as the desired wavelength through temperature control of the laser diode. In addition, it was confirmed that the four wavelengths, combined through an optical coupler, were switched at a speed of 500 μs using an optical switch.

## 3 Fast and reliable phase determination in multiwavelength interferometry

In MWI, extracted phase maps are wrapped from the reference plane, and the number of wraps depends on the wavelengths. Therefore, phase unwrapping in multiwavelength interferometry requires identifying the correct number of wraps for each wavelength to accurately determine the absolute distance. This process corresponds to determining the fringe order for step heights greater than half of the wavelength. Phase unwrapping in multiwavelength interferometry is categorized into beat wavelength methods [39], Chinese Remainder Theorem [40], and the excess fraction method (EFM) [41], [42]. The beat synthetic wavelength method computes the synthetic phase of the selected synthetic wavelength and infers the surface based on it. The beat synthetic wavelength method is time-efficient but strict control over wavelength stability is essential. The Chinese Remainder Theorem assumes that the extracted phase corresponds to the remainders obtained by dividing the OPD by each wavelength, requiring both the wavelengths and the OPD to be integers. The EFM calculates the residuals between the calculated and measured fractions, offering high reliability but requiring significant computational resources, making it less time-efficient.

### 3.1 Recursive excess fraction method

The EFM determines the integer part by comparing the calculated and measured fractional parts of multiple wavelengths. The integer part of the reference wavelength is estimated based on empirical ranges or prior knowledge of the target length. The first wavelength, *λ*_1_, is used as the reference. The fractional parts of the other wavelengths, denoted as *e*_*cal,i*_ can be calculated by dividing the length, corresponding to the estimated integer *m*_*t*_ by each wavelength. An inaccurate estimation of the integer part results in the residual Δ*e*_*i*_, which represents the difference between the calculated and measured fractional parts. The root mean square of residuals (RMSR) across all wavelengths is represented as *R*, where:

(11) $$e_{\mathrm{cal,i}}=E\left[2\cdot L/\lambda_{i}\right]=E\left[\left(m_{t}+e_{1}\right)\frac{\lambda_{1}}{\lambda_{i}}\right]$$

(12) $$R\left(m_{t}\right)=\sqrt{\overset{n}{\underset{i=2}{\sum }}{\left[e_{i}-e_{\mathrm{cal,i}}\right]}^{2}}$$

**Figure 4:**
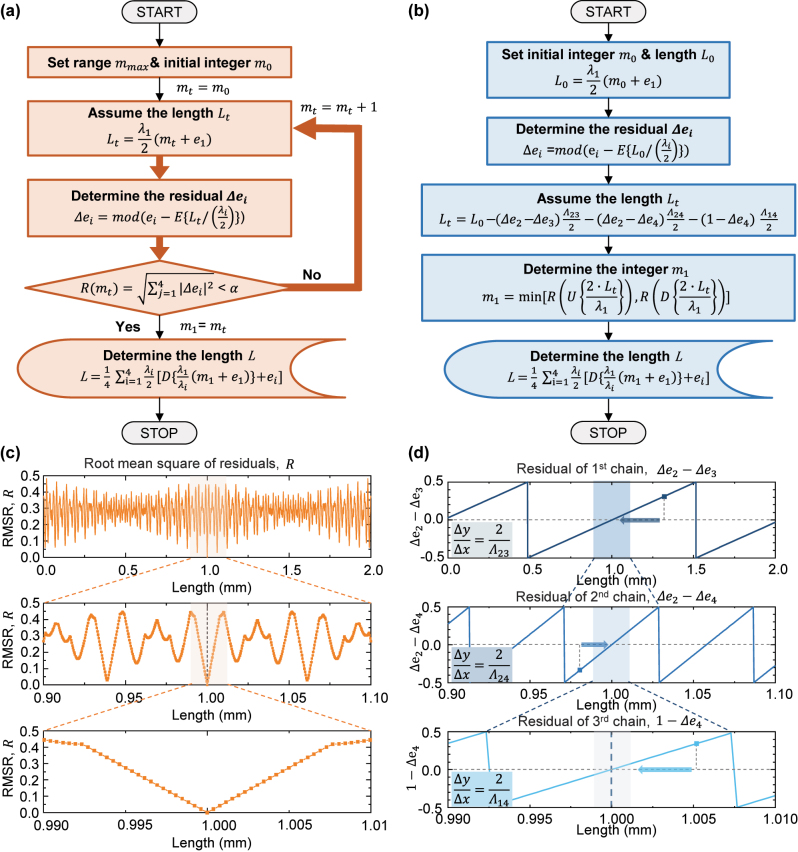
Phase detection algorithm to overcome 2π phase ambiguity for measuring large-stepped discontinuous surfaces. (a) Flowchart of EFM with iterative calculations and (b) flowchart of SEFM. (c) Simulation showing the RMS of residuals in EFM as a function of length. (d) Simulation illustrating the sequential approach in SEFM, based on the residuals of synthetic wavelengths. The function *E*[∙] returns the fractional part, while *D*[∙] and *U*[∙] returns the floored and ceiling integer values, respectively. The mod[∙] function provides values after modular correction with wrap adjustment.

*e*_*cal,i*_ represents the calculated fractional phase for the *i*-th wavelength, taking the first wavelength as the reference, while *e*_*i*_ denotes the fractional phase measured at the *i*-th wavelength. If the estimated integer corresponds to the true value, the residuals for all wavelengths approach zero. The function *E*[∙] returns the fractional part and *D*[∙] returns the integer obtained by flooring the value. Therefore, to find the minimum *R* or until it is less than the tolerance *α*, the integer *m*_*t*_ is iteratively replaced with the next integer and re-evaluated. Figure 4a illustrates the flowchart of EFM.

This approach, however, presents two disadvantages. Firstly, a strict tolerance is necessary to determine a unique solution. Given the phase noise induced by the system and environmental factors, generalizing the tolerance value is challenging, and an incorrect choice of tolerance can lead to multiple solutions. Even with an appropriately chosen tolerance, unexpected phase noise may result in multiple solutions, ultimately compromising reliability and preventing accurate solution selection. Secondly, the method is time-consuming as it necessitates repetitive calculation and comparison of $R\left(m_{t}\right)$ for each wavelength and tolerance with respect to the altered integer.

### 3.2 Nonrecursive sequential excess fraction method

To enhance the reconstruction of a 3D profile with greater speed without compromising reliability, we developed a sequential method of excess fraction (SEFM), as depicted in Figure 4b. Similar to EFM, this approach uses residuals of specific wavelengths to identify the integer part that minimizes the *R*-value. Significantly, this involves the strategic selection of synthetic wavelengths based on the phase uncertainty criteria outlined in Section 2.1, which facilitates stable and rapid convergence of the solution. As observed in Figure 4c, the RMSR in EFM displays periodicity of various beats of synthetic wavelengths. The simulation presents the RMSR values as a function of length when the surface is located at 1 mm, utilizing preselected wavelengths based on phase uncertainty criteria. When the integer part, *m*_*t*_, corresponding to 1 mm length is substituted, the RMSR value is observed to approaches zero.

Similarly, the residual of the synthetic wavelength also shows periodicity at half the wavelength period, with the slope generated at half the synthetic wavelength. This slope can be effectively utilized to rapidly identify the point of convergence. Initially, an arbitrary distance *L*_0_ within NAR is substituted to find the residuals of synthetic wavelengths, Δ*e*_2 _and Δ*e*_3_, at the nearest wavelengths. Subsequently, as demonstrated in upper part in Figure 4d, the difference between Δ*e*_2_ and Δ*e*_3_, along with the slope of 2/Λ_23_, is used to compute the *x*-intercept value, thereby facilitating the convergence process. After convergence, the phase uncertainty range is replaced by a distance uncertainty range, as shown in Equation (13).

(13) $$\begin{array}{ll} & L_{0}-\frac{\Lambda_{23}\cdot \left(\Delta e_{2}-\Delta e_{3}\right)}{2}-\frac{\Lambda_{23}\cdot \left[u\left(\Delta e_{2}-\Delta e_{3}\right)\right]}{2}<L_{t}\\ & \;<L_{0}-\frac{\Lambda_{23}\cdot \left(\Delta e_{2}-\Delta e_{3}\right)}{2}+\frac{\Lambda_{23}\cdot \left[u\left(\Delta e_{2}-\Delta e_{3}\right)\right]}{2} \end{array}$$

If the range of uncertainty is less than half the period of the next smaller synthetic wavelength (Λ_24_),

(14) $$\Lambda_{23}u\left(\Delta e_{2}-\Delta e_{3}\right)<\frac{\Lambda_{24}}{2}$$

By substituting Equation (13) into Equation (14), the convergence process can be performed again within a narrow distance range using a smaller synthetic wavelength.

(15) $$\begin{array}{ll} & L_{0}-\frac{\Lambda_{23}\left(\Delta e_{2}-\Delta e_{3}\right)}{2}-\frac{\Lambda_{24}}{4}<L_{t}\\ & \;<L_{0}-\frac{\Lambda_{23}\left(\Delta e_{2}-\Delta e_{3}\right)}{2}+\frac{\Lambda_{24}}{4} \end{array}$$

Afterward, this process can be carried out at the next synthetic wavelength (Λ_14_), significantly narrowing the range of length and integer as given in Equations (16) and (17), respectively.

(16) $$L_{0}-\frac{\Lambda_{14}\cdot \Delta e_{4}}{2}-\frac{\lambda_{1}}{4}<L_{t}<L_{0}-\frac{\Lambda_{14}\cdot \Delta e_{4}}{2}+\frac{\lambda_{1}}{4}$$

(17) $$\left[m_{0}-\frac{\Lambda_{14}\cdot \Delta e_{4}}{\lambda_{1}}-0.5\right]\leq m_{1}\leq \left[m_{0}-\frac{\Lambda_{14}\cdot \Delta e_{4}}{\lambda_{1}}+0.5\right]$$

The *m*_1_ value that satisfies these conditions and has the minimum RMSR is obtained, and by calculating the distance (*L*_1_) at each wavelength based on *m*_1_, the final distance value is obtained through the averaging. The distance calculated at each wavelength using *m*_*i*_ is averaged to obtain the final distance value. Finally, as shown in Figure 4c, the proposed method allows for accurate results through sequential calculation, avoiding the need for conventional recursive calculation. Accordingly, it offers a significant advantage in terms of 3D reconstruction time, which increases proportionally with the number of pixels.

### 3.3 Reliability and speed performance evaluation

**Figure 5:**
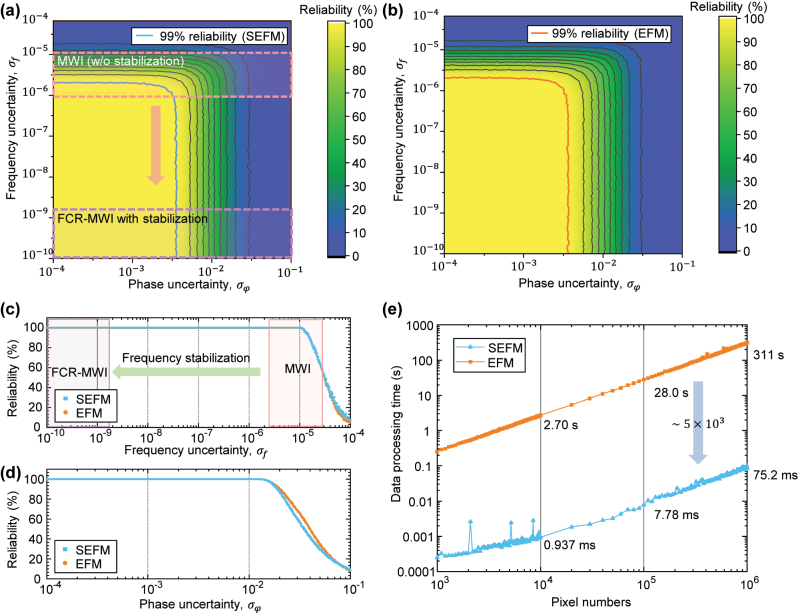
Performance analysis of the sequential EFM and conventional EFM under varying noise conditions. (a) Reliability map of SEFM and (b) EFM under phase and frequency uncertainty. (c) Reliability of both methods with respect to frequency uncertainty and (d) with respect to phase uncertainty. (e) Comparison of data processing times as a function of the number of pixels.

The magnitude of deviation caused by phase unwrapping algorithms depends on the specific algorithm employed. An incorrect determination of the integer part can lead to a deviation of at least half a wavelength, which significantly impacts measurement precision regardless of the exact magnitude of the deviation. Therefore, to evaluate the accuracy of phase unwrapping through simulations, it is appropriate to assess the probability of failing to correctly determine the integer part under noise. Simulations were conducted to evaluate the reliability of the phase unwrapping. Figure 5a and b illustrate the reliability of SEFM and EFM based on the number of solutions extracted from 10,000 cases, with varying magnitudes of Gaussian white noise in phase and frequency. Both EFM and SEFM exhibit reduced reliability as phase or frequency uncertainty increases. Figure 5c evaluates reliability with Gaussian noise affecting only frequency, while Figure 5d addresses noise affecting only phase, showing that phase noise exceeding 10^−2^ significantly degrades measurement reliability.

In Figure 5c, frequency uncertainties in unstabilized multiwavelength interferometry range between 10^−4^ and 10^−6^, and since actual measurements include phase uncertainty due to measurement noise, neither SEFM nor EFM maintains high reliability within this range. Lower reliability indicates an increased likelihood of local minimum solutions, critically impacting measurement precision. Stabilizing the light source with optical combs reduces frequency uncertainty to below 10^−10^, enabling reliable measurements. This was confirmed by experimental data presented in Section 4, Figure 7b. Figure 5e shows the step height reconstruction times as a function of pixel count. The EFM required 315.3 s for 1 Mpixel, whereas the SEFM achieved a reconstruction time of approximately 75.2 ms under the same conditions, demonstrating a speed improvement of over 5 × 10^3^ times. This speed advantage remains consistent as the pixel count increases.

## 4 3D surface measurement and reconstruction

### 4.1 3D profile measurement of standard sub-mm and μm-level height samples

**Figure 6:**
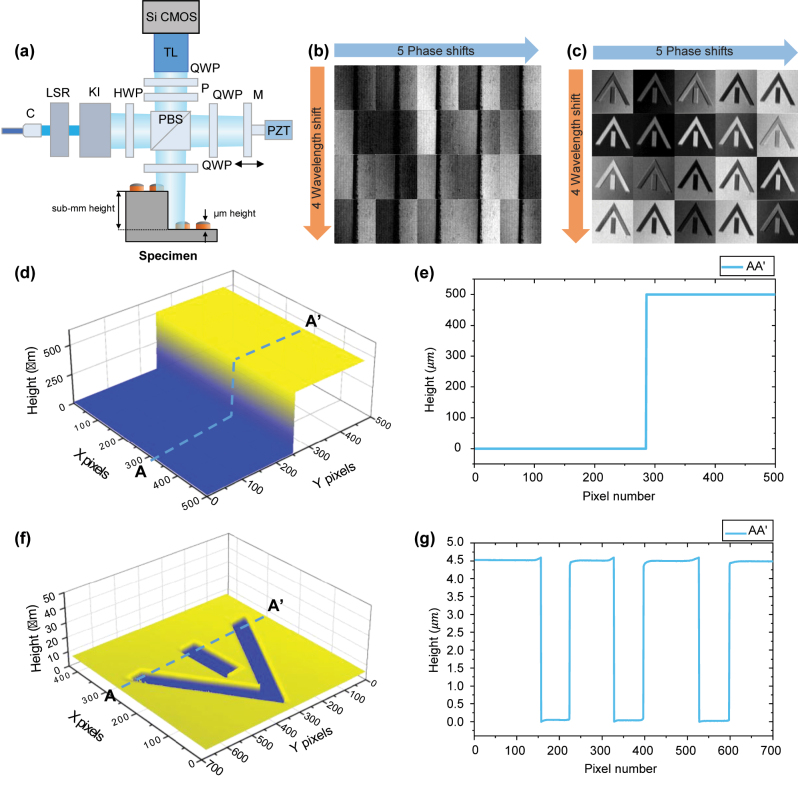
Stepped discontinuous surface measurement using a multiwavelength interferometer based on frequency-comb-referenced laser diodes. (a) Configuration of PZT-based polarization Twyman–Green interferometry, (b) and (c) interference fringes as a function of wavelength and phase shift for 3D reconstruction, (d) and (f) reconstructed surface profiles, and (e) and (g) cross sections of the 500 μm step height specimen and the 4.5 μm height standard specimen, respectively. LSR: laser speckle reducer module, KI: Köhler illumination module, HWP: half-wave plate, QWP: quarter-wave plate, PBS: polarization beam splitter, P: polarizer, C: collimator, TL: telecentric lens.

As shown in Figure 6a, a Twyman–Green interferometer with polarization components was utilized. A laser speckle reducer (LSR) module was incorporated to reduce specular reflection from the rough surfaces of the measurand. Köhler illumination (KI) module was designed to provide uniform illumination of the sample. A telecentric lens was employed to eliminate distortion during the measurement of the bump’s top surface. The system utilized a 12 M pixel camera (Vieworks VC-12MX-M330, 4,096 × 3,072 pixels, pixel size: 5.5 µm × 5.5 µm) coupled with a ×1.6 magnification telecentric lens, resulting in a total field of view (FOV) of 14.0 × 10.4 mm^2^. The lateral resolution was calculated to be 3.45 µm × 3.45 µm and was verified through a resolution target measurement. The depth of field (DOF) was determined by the numerical aperture (NA), and with a low NA (=0.098), the system achieved a high DOF of 0.19 mm.

The acquisition time for getting a single interferogram by camera is 3.3 ms (330 Hz). And we capture 20 interferograms to accommodate 4 wavelengths and 5 phase shifts, resulting in a total acquiring time of 109.5 ms. To achieve rapid measurements over a wide area, we used a 12 M pixel CMOS with a sensor size of 22.4 mm × 16.9 mm (5.5 µm × 5.5 µm, pixel size), employing a CoaXPress interface based on 8 channels with a maximum data transmission speed of 50 Gbps. By reducing the measurement area (regions of interest, ROI) with increasing the light intensity, the measurement speed could be improved by more than 10 times.

To verify the resolution and extended measurement range of the system, a 500 μm steel gauge block (Mitutoyo) and a specimen with a step of 4.428 μm and an uncertainty of 0.058 μm (VLSI standards, SHS-4.5QC-G2) were measured. Figure 6b and c show the phase-shifted interference patterns at four different wavelengths. The phase was extracted using the phase-shifting algorithm, and the 3D profile was reconstructed using SEFM. The proposed method takes 75.2 ms to process 1 Mpx and 891 ms for a total of 12 Mpx, corresponding to 75.2 ns per pixel. In the case of a gauge block measurement, as shown in Figure 6d, it was measured at a step height of 500.008 µm. Figure 6g shows the cross section of Figure 6f, the height of 4.428 μm was measured to be 4.411 μm, and the deviation was 65 nm. This confirms that the measurement fell within the uncertainty range provided for the sample. The bat-wing effect observed at the discontinuous edges appears to have been mitigated through vertical range limiting and trimming, although it still remains to some extent.

### 4.2 Repeatability in standard deviation and in Allan deviation

**Figure 7:**
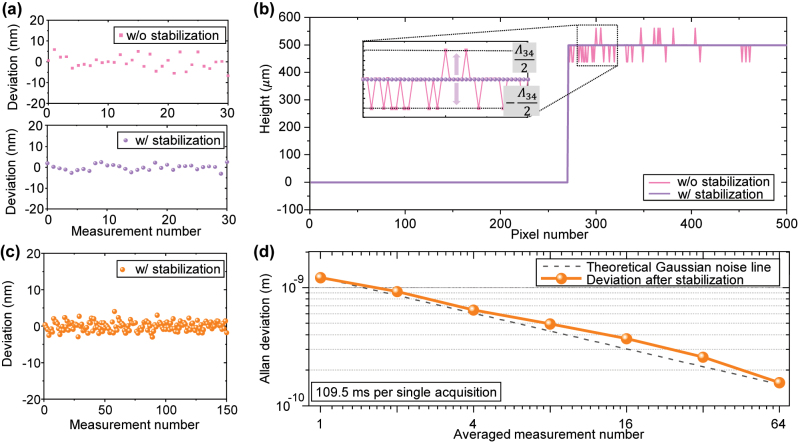
Repeatability analysis of proposed multiwavelength interferometer. (a) 30 consecutive measurements (b) reconstructed cross section on 500 μm height specimen w/and w/o laser diode stabilization. (c) 150 consecutive measurements. (d) Allan deviation at a single point on 500 μm height specimen.

To evaluate the precision of the proposed system, the deviations observed in consecutive measurements were analyzed. Surface repeatability is significantly affected by vibrations and environmental disturbances. Recursive methods, such as the 4 + 1 bucket phase-shifting technique, can be employed to mitigate these effects. However, these methods fail to effectively compensate for low-frequency drift, which can degrade repeatability. To address this issue, the step height repeatability in this study was evaluated by calculating the difference between points on the upper and lower surfaces. Specifically, the step height repeatability was calculated by subtracting the measurement points on the bottom surface from those on the top surface to reduce the impact of low-frequency drift. Figure 7a shows the extracted phase values before and after referencing to frequency comb at a single point on a 500 μm height specimen. The standard deviation of the extracted height, measured 30 times and amounting to 2.58 nm (0.0066 *λ*), was obtained before stabilization. When an EFM is applied using a light source with this precision, deviation tend to increase due to the local solutions, making it difficult to determine the correct integer part due to multiple solutions, as depicted by the pink line in Figure 7b. However, after stabilization with the SEFM, the standard deviation of height improved to 1.35 nm (0.0034 *λ*). Consequently, as indicated by the purple line in Figure 7b, this improvement allowed the measurement range to be stably extended to 1 mm without encountering the issue of local solutions. The unexpected “jumped” local solutions occur at half of the synthetic wavelength away from the true value. This issue becomes more pronounced as the vertical distance from the reference plane increases.

According to the uncertainty analysis, the distance uncertainty for a 500 µm measurement was found to be 2.37 fm due to wavelength uncertainties and 8.5 pm due to refractive index uncertainties. However, the repeatability over 30 measurements was 1.35 nm, which is about 10^5^ times larger than distance uncertainty caused by the wavelength uncertainties. These results indicate that the high-precision characteristics of the optical comb are significantly constrained by the inherent vibration sensitivity of the phase-shifting interferometer.

To further evaluate the precision of step height repeatability under the influence of random noise in time-series measurements, Allan deviation was assessed as illustrated in Figure 7c and d [43], [44], focusing on averaging out errors caused by environmental uncertainty factors such as vibrations and disturbances. Allan deviation analysis demonstrates that the multiwavelength interferometer can reach 0.258 nm precision with averaging over 32 measurements. The Allan deviation results show that as the number of averaged data points increases, the averaging effect becomes stronger, leading to a decreasing in Allan deviation. However, if more data is collected and there are disturbances with extremely slow frequencies (half of the averaging time), Allan deviation is expected to increase again. In other words, while averaging can improve precision in non-common-path interferometer systems, it also limits the measurement time.

### 4.3 3D profile measurement of microbumps with 30-μm and 10-μm heights

**Figure 8:**
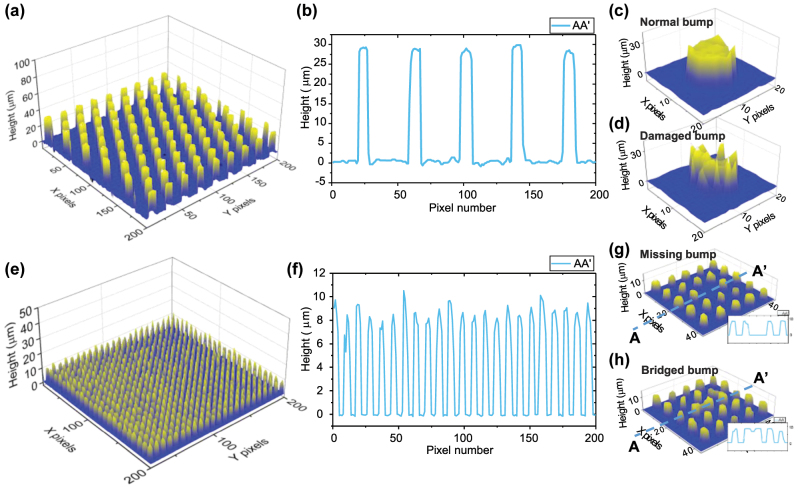
Microbump measurement. (a) Reconstructed 3D profile and (b) cross section of 30 μm height microbump. 3D profile of (c) normal bump and (d) damaged bump. (e) Reconstructed 3D profile and (f) cross section of 10 μm height microbump. 3D profile of defects; (g) missing bump and (h) bridged bump.

Microbumps with 30 µm and 10 µm height were manufactured using an alloy with a Sn-to-Pb ratio of 95:5, which is commonly used in industry. Through the patterned PR coating, SnPb was grown cylindrical, and the PR and seed metal were removed through etching. The sample was then placed on a quartz substrate and vacuum annealed at 800 °C for 40 minutes. Argon gas of 99.9999 % purity was used during the annealing process to prevent oxidation (SnO_2_). Figure 8a and e show the 3D reconstructed profiles using the proposed multiwavelength interferometer and algorithm in this study, and Figure 8b and f represent cross sections of Figure 8a and e, respectively. Furthermore, as demonstrated in Figure 8c and d, both normal bump and damaged bump were accurately measured. Additionally, as seen in Figure 8g and h, that other types of microbump defects, such as missing and bridged bumps, were also accurately measured.

## 5 Conclusions

In this study, we proposed a frequency-comb-referenced multiwavelength interferometry for high-speed and high-precision measurement of 3D surfaces profile of advanced semiconductor packaging products. By phase-locking four continuous-wave tunable lasers to the frequency comb of a femtosecond laser stabilized to an Rb clock and optical clock, we realized a set of highly stabilized light sources with fractional stability of 4.77 × 10^−12^ with optical power of tens of mW per channel. This high stability of the light source improves the measurement precision to nm-level and then extends the effective measurement range by eliminating the possibility of local solutions. We also proposed a sequential exact fraction method, which accelerated the absolute phase detection process by 5,000 times without compromising reliability. To demonstrate the multiscale 3D measurement in semiconductor packaging, step heights over 500 µm and microbumps were measured. The step height measurement results were within the uncertainty range, and defects on the microbumps generated during the reflow process were also inspected. The system required 109.5 ms to acquire interferograms, and the phase detection algorithm took 75.2 ms for 1 Mpixel, achieving a measurement precision of 0.258 nm with 32-measurement averaging. By locking to a frequency comb of SI time/frequency standard, the measurement results in the entire production line can be directly traced to the national standards, eliminating the need for periodic calibration. These advancements suggest that high-precision and high-speed shape measurement using this frequency-comb-referenced multiwavelength interferometer can be applied to in-line process monitoring in next-generation advanced semiconductor packaging.
